# Metabolic risk factors and risk of Covid-19: A systematic review and meta-analysis

**DOI:** 10.1371/journal.pone.0243600

**Published:** 2020-12-15

**Authors:** Bahram Moazzami, Shahla Chaichian, Amir Kasaeian, Shirin Djalalinia, Meisam Akhlaghdoust, Masoud Eslami, Behrooz Broumand

**Affiliations:** 1 Pars Advanced and Minimally Invasive Medical Manners Research Center, Pars Hospital, Tehran, Iran; 2 Hematology, Oncology and Stem Cell Transplantation Research Center, Tehran University of Medical Sciences, Tehran, Iran; 3 Digestive Diseases Research Center, Digestive Diseases Research Institute, Tehran University of Medical Sciences, Tehran, Iran; 4 Development of Research and Technology Center, Deputy of Research and Technology, Ministry of Health and Medical Education, Tehran, Iran; 5 Non-Communicable Diseases Research Center, Endocrinology and Metabolism Population Sciences Institute, Tehran University of Medical Sciences, Tehran, Iran; 6 Tehran University of Medical Sciences, Tehran, Iran; University of South Carolina, UNITED STATES

## Abstract

**Objective:**

Based on the epidemiologic findings of Covid-19 incidence; illness and mortality seem to be associated with metabolic risk factors. This systematic review and meta-analysis aimed to assess the association of metabolic risk factors and risk of Covid-19.

**Methods:**

This study was designed according to PRISMA guidelines. Two independent researchers searched for the relevant studies using PubMed, Web of Science, Cochrane Library, and Scopus. The search terms developed focusing on two main roots of “Covid-19” and “metabolic risk factors”. All relevant observational, analytical studies, review articles, and a meta-analysis on the adult population were included in this meta-analysis. Meta-analysis was performed using the random effect model for pooling proportions to address heterogeneity among studies. Data were analyzed using STATA package version 11.2, (StataCorp, USA).

**Results:**

Through a comprehensive systematic search in the targeted databases we found 1124 papers, after running the proses of refining, 13 studies were included in the present meta-analysis. The pooled prevalence of obesity in Covid-19 patients was 29% (95% CI: 14–47%). For Diabetes and Hypertension, these were 22% (95% CI: 12% 33%) and 32% (95% CI: 12% 56%), respectively. There was significant heterogeneity in the estimates of the three pooled prevalence without any significant small-study effects. Such warning points, to some extent, guide physicians and clinicians to better understand the importance of controlling co-morbid risk factors in prioritizing resource allocation and interventions.

**Conclusion:**

The meta-analysis showed that hypertension is more prevalent than obesity and diabetes in patients with Covid-19 disease. The prevalence of co-morbid metabolic risk factors must be adopted for better management and priority settings of public health vaccination and other required interventions. The results may help to improve services delivery in COVID-19 patients, while helping to develop better policies for prevention and response to COVID-19 and its critical outcomes.

## 1. Introduction

From December 2019, coronavirus disease −2019 (COVID-19) that was reported from Wuhan-China, quickly became a global pandemic. Due to the speed of the spread and the severity of individual and social complications and consequences, pandemic unexpectedly has resulted in a significant strain on healthcare systems [1, 2].

Because of the unknown complex nature of the disease, there are many doubts about the related and predisposing factors. Initially, the disease with prominent symptoms of the pulmonary respiratory system was thought to be more severe in the elderly [1, 3]. At the same time, in a very short period of time, the virus surprised the health care workers with the speed of changing the patterns of clinical symptoms and ddifferent impact in diverse target groups [4, 5].

Based on the epidemiologic findings of COVID-19 incidence, the severity of illness, and mortality seem to be associated with multiple comorbidities such as diabetes, hypertension, and cardiovascular disease [1, 6]. Some studies revealed that severe COVID-19 patients have a higher incidence of diabetes and hypertension than milder forms [7, 8]. There are scattered researches on the association between obesity and overweight and the risk of disease [6]. There is also different evidence from the results of treatment protocols and intensive care unit outcomes that emphasizes the association between metabolic risk factors and the consequences of different interventions [1, 8, 9].

Policymakers and health managers need reliable information to help them make accurate decisions and take timely and effective actions [5]. Comprehensive analysis of applied research can help policymakers to make decisions on screening plans for high-risk groups. High priority should be given to patients with COVID-19 infection who also simultaneously suffer from metabolic risk factors. This approach may even influence clinical decisions such as tracking of diabetics or hypertensive who are registered as COVID-19 patients or even how to distribute and allocate inpatient, emergency facilities, and intensive care. Despite or the priority of the problem, there is an evident gap in practical comprehensive documents on the association of metabolic risk factors and Covid-19 [1, 10, 11]. Therefore, to get more convincing results, we aimed to conduct a systematic review addressed the association between the presence of the metabolic risk factors and the risk of Covid-19 incidence in the adult population.

## 2. Methods

Aimed to assess the association between metabolic risk factors and Metabolic syndrome (MetS) and Covid-19 we followed the Preferred Reporting Items for Systematic Reviews and Meta-Analyses (PRISMA) statement [12].

### 2.1. Data source and search strategy

Based on developed protocol, systematically, all available related papers searched from comprehensive international databases of PubMed and NLM Gateway (for MEDLINE), Institute of Scientific Information (ISI), Cochrane library, and Scopus, from inspection of disease to 7 Jun 2020. Regardless of the time of the study or the date of publication of the results and the language of the published articles, all relevant studies were included in the processes of refinement.

The main roots for the development of search strategies formed on the basis of “Covid-19” and components of “metabolic risk factors”. The components of metabolic risk factors were: obesity/overweight; Diabetes (T2DM); hypertension and hypercholesterolemia. We also specifically searched for relevant studies on metabolic syndrome (MetS). In completing this syntax; all related terms included according to this main strategy (Table 1). In addition, the reference lists and cited articles of the included papers were searched manually to find any additional papers.

**Table 1:** Search strategy.

PubMed
((((covid[Title/Abstract]) OR "COVID-19"[Title/Abstract]) OR coronavirus[Title/Abstract])) AND ((((((("Metabolic Syndrome"[Mesh]) OR (((((((((((((((((((("X Syndrome"[Title/Abstract]) OR "Syndrome X"[Title/Abstract]) OR "Insulin Resistance"[Title/Abstract]) OR Diabetes[Title/Abstract]) OR "HDL"[Title/Abstract]) OR "LDL"[Title/Abstract]) OR "hyper lipid"[Title/Abstract]) OR "TG"[Title/Abstract]) OR "hyper cholesterol"[Title/Abstract]) OR cardiometabolic[Title/Abstract]) OR cardio-metabolic[Title/Abstract]) OR "cardio metabolic"[Title/Abstract]) OR "blood pressure"[Title/Abstract]) OR systolic[Title/Abstract]) OR diastolic[Title/Abstract]) OR triglyceride[Title/Abstract]) OR cholesterol[Title/Abstract]) OR overweight[Title/Abstract]) OR waist to hip ratio [Title/Abstract]) OR waist- hip ratio [Title/Abstract]) OR waist circumference [Title/Abstract]) OR obesity[Title/Abstract]) OR "BMI"[Title/Abstract])) OR "Obesity, Abdominal"[Mesh]) OR (("Body Mass Index"[Mesh]) OR "Overweight"[Mesh])) OR "Diabetes Mellitus"[Mesh]) OR "Cholesterol, LDL"[Mesh]) OR (((("Fasting plasma glucose"[Title/Abstract]) OR "Fasting Blood glucose"[Title/Abstract]) OR "FPG"[Title/Abstract]) OR "FBS"[Title/Abstract]))
Scopus
( (TITLE-ABS-KEY (covid) OR TITLE-ABS-KEY (coronavirus) ) ) AND ( ( ( (TITLE-ABS-KEY ("Metabolic Syndrome") OR TITLE-ABS-KEY ("Syndrome X") OR TITLE-ABS-KEY ("X Syndrome") OR TITLE-ABS-KEY ("Insulin Resistance") OR TITLE-ABS-KEY ("Diabetes") OR TITLE-ABS-KEY ("HDL") OR TITLE-ABS-KEY ("LDL") OR TITLE-ABS-KEY ("hyper lipid") OR TITLE-ABS-KEY ("TG") ) ) OR ( (TITLE-ABS-KEY ("hyper cholesterol") OR TITLE-ABS-KEY ("cardiometabolic") OR TITLE-ABS-KEY ("cardio metabolic") OR TITLE-ABS-KEY ("cardio-metabolic") OR TITLE-ABS-KEY ("blood pressure") OR TITLE-ABS-KEY (systolic) OR TITLE-ABS-KEY (diastolic) OR TITLE-ABS-KEY (triglyceride) OR TITLE-ABS-KEY (cholesterol) ) ) ) OR ( (TITLE-ABS-KEY ("overweight") OR TITLE-ABS-KEY (obesity) OR TITLE-ABS-KEY ("BMI") OR TITLE-ABS-KEY ("Body mass index") OR TITLE-ABS-KEY (" waist to hip ratio ") OR TITLE-ABS-KEY (" waist- hip ratio ") OR TITLE-ABS-KEY (" waist circumference ") OR TITLE-ABS-KEY ("Fasting plasma glucose") OR TITLE-ABS-KEY ("Fasting blood glucose") OR TITLE-ABS-KEY ("FPG") OR TITLE-ABS-KEY ("FBS") ) ) ) AND (EXCLUDE (DOCTYPE, "le") OR EXCLUDE (DOCTYPE, "ed") OR EXCLUDE (DOCTYPE, "sh") OR EXCLUDE (DOCTYPE, "cp") OR EXCLUDE (DOCTYPE, "ch") OR EXCLUDE (DOCTYPE, "er") OR EXCLUDE (DOCTYPE, "bk") ) AND (EXCLUDE (SUBJAREA, "ENVI") OR EXCLUDE (SUBJAREA, "COMP") OR EXCLUDE (SUBJAREA, "SOCI") OR EXCLUDE (SUBJAREA, "ARTS") OR EXCLUDE (SUBJAREA, "ENGI") OR EXCLUDE (SUBJAREA, "MATE") OR EXCLUDE (SUBJAREA, "MATH") OR EXCLUDE (SUBJAREA, "PHYS") ) AND (EXCLUDE (DOCTYPE, "no") ) AND (EXCLUDE (SRCTYPE, "k") )
ISI/WOS
TOPIC: (covid) OR TOPIC: (coronavirus) Indexes = SCI-EXPANDED, SSCI, A&HCI, CPCI-S, CPCI-SSH, BKCI-S, BKCI-SSH, ESCI, CCR-EXPANDED, IC Timespan = All years TOPIC: ("Metabolic Syndrome") OR TOPIC: ("X Syndrome") OR TOPIC: ("Insulin Resistance") OR TOPIC: (Diabetes) OR TOPIC: ("HDL") OR TOPIC: ("Blood Glucose") OR TOPIC: ("LDL") OR TOPIC: ("hyper lipid") OR TOPIC: ("TG") OR TOPIC: (cholesterol) OR TOPIC: (cardiometabolic) OR TOPIC: ("cardio metabolic") OR TOPIC: ("cardio-metabolic") OR TOPIC: ("blood pressure") OR TOPIC: (systolic) OR TOPIC: (diastolic) OR TOPIC: (triglyceride) OR TOPIC: (overweight) OR TOPIC: (obesity) OR TOPIC: (waist to hip ratio) OR TOPIC: (waist-hip ratio) OR TOPIC: (waist circumference) OR TOPIC: ("LDL") OR TOPIC: ("BMI") OR TOPIC: ("Body mass index") OR TOPIC: ("Fasting plasma glucose") OR TOPIC: ("blood glucose") OR TOPIC: ("FPG") OR TOPIC: ("FBS") Indexes = SCI-EXPANDED, SSCI, A&HCI, CPCI-S, CPCI-SSH, BKCI-S, BKCI-SSH, ESCI, CCR-EXPANDED, IC Timespan = All years

### 2.2. Inclusion and exclusion criteria

This systematic review included all relevant observational, analytical studies, review articles, and meta-analysis on the adult population. There was not any restriction in terms of countries, ethnicity, and gender of the participants. There was no time limit for studying or publishing articles or the language of publication of results.

We excluded non-human-based studies or those with duplicate citations. The studies that focused on non-generalized specific sub-group populations were also excluded. Studies with duplicate citations were excluded. In the situation of multiple publications on the same population, only the largest study or the main source of data was included. Moreover, if there were any low-quality papers that had no eligible data for collection it was deleted.

### 2.3. Study selection

**Fig 1:**
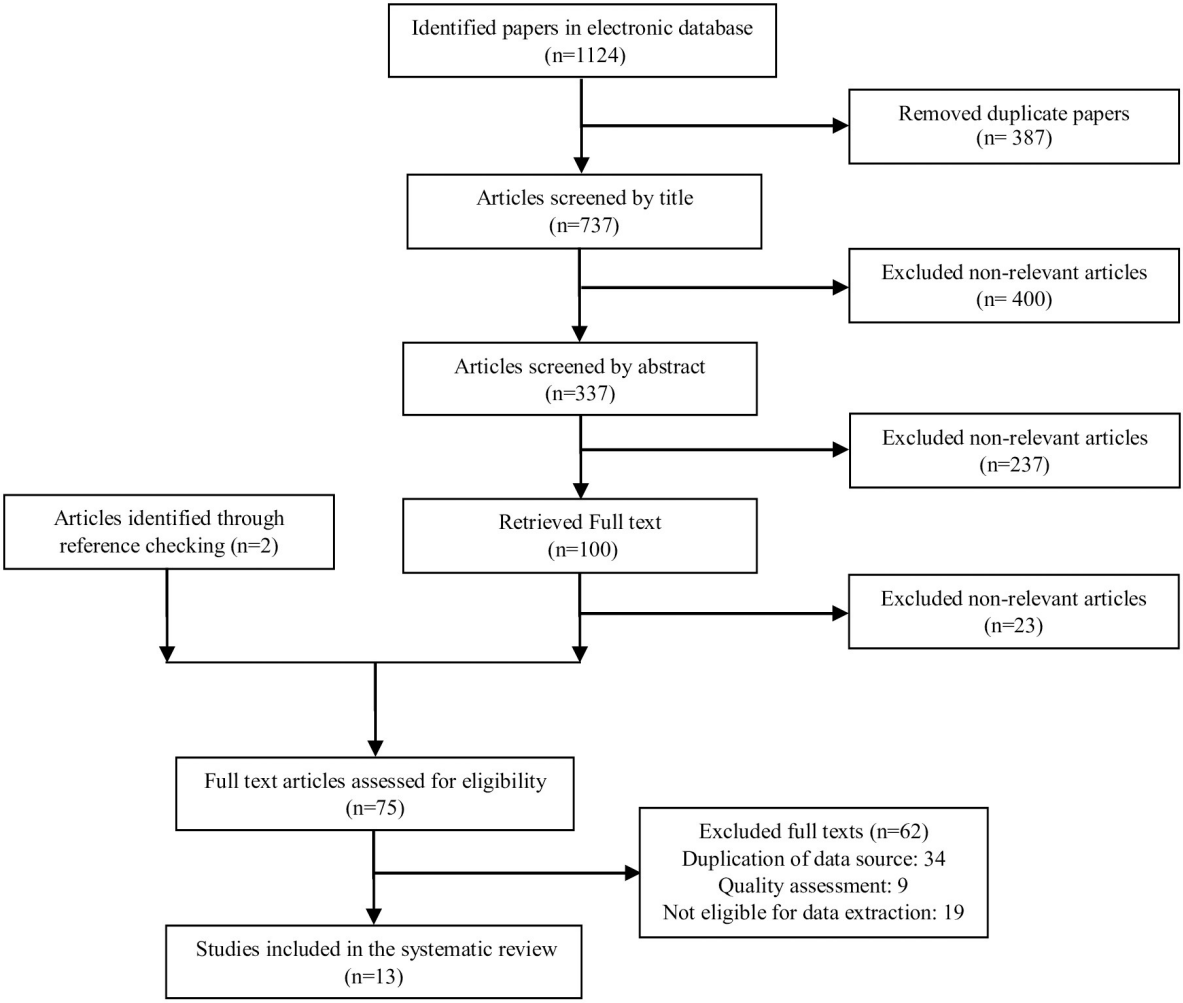
Papers search and review flowchart for selection of primary study.

After conducting the searches, all of the results saved into the Endnote software. All records refined for three sequential steps of relevancy assessment, which was run according to the evaluation of titles, abstracts, and full texts, respectively. Remained papers evaluated for their quality (Fig 1).

The quality assessment of remained papers conducted, independently, by two research experts. The quality of study design, sampling strategy, and measurement quality assessed based on Consort 2010 check list and Jadad scoring for clinical trials and STROBE quality assessment tools for observational studies (cohort, case-control, and cross-sectional studies).

### 2.4. Data extraction

For remained relevant papers, based on the PRISMA 2009 statement checklist, the quality assessment of the studies evaluated, and using a customized form, relevant data were independently extracted from eligible papers. The following data were extracted: first author, year of publication, country, research design, sources of the patients, types of the study population, sample size, the patient’s demographic data, the status of metabolic risk factors by types of diagnostic indicators and specific units, Covid-19 and diagnostic criteria, recommendations, and specific practical points.

All the sequential process of systematic search, refinement of papers, quality assessment and data extraction were followed independently by two independent researchers (Kappa statistic for agreement for quality assessment; 0.92). Probable discrepancy resolved through referencing the principal investigator’s opinions’.

### 2.5. Statistical analysis

Meta-analysis was performed by means of the STATA package version 11.2 (Stata Corporation, College Station, TX, USA) to combine the prevalence of all studies and conclude the pooled prevalence and its 95% confidence interval (CI) using a random effect model for pooling proportions [13]. Forest plots were drawn showing the variation of the prevalence among all studies together with the pooled measure [14]. Between-study heterogeneity was evaluated by the Cochran’s Q test and the I2 statistic [15]. A fixed-effects model was applied (P > 0.05 or I2 < = 50%). Otherwise, a random-effect model was applied (P < 0.05 or I2 greater than 50%). Presence of small-study effects (ie, whether smaller studies tend to give substantially larger estimates of effect size compared with larger studies) was assessed using the Egger’s regression asymmetry test [16, 17]. All p-values were two-tailed.

### 2.6. Ethical considerations

The present study has been approved by the ethical committee of non-governmental Pars Advanced and Minimally Invasive Medical Manners Research Center of Pars Hospital, Tehran, Iran. All of the included studies in our review would be cited in all reports and all extracted publications of our study. For more required information we contacted the corresponding author.

## 3. Results

Running the search strategy initially led to a total of 1124 articles. After removing duplicates, checked the relevancy of titles and abstracts, and reviewing the full-texts, 47 studies remained for analysis (Fig 1). Through rechecking the records, we found that thirty studies related to China and nine studies related to the USA. In China, there were one nation-wide and 29 local studies in which we were to select that one national-wide study and 3 other local studies and omitted the other 26 local studies due to time overlap with those four main selected ones.

In the USA, on the other hand there, there were nine studies that 3 of them were national-wide and 6 of them were local. From those 3 national-wide ones we chose the largest study that belongs to CDC and all the other studies were again omitted due to time overlap. Finally, thirteen studies related to 10 countries remained in the final analysis. Besides, studies related to Italy and Spain was nation-wide and the study related to the UK was a region-wide study. Thus, we select the most comprehensive sources of data. The features of the 13 studies were shown in Table 2.

**Table 2:** Main characteristics of included studies in the meta-analysis.

No	Author	Country, Year	Study Design	Study Scope	Sample Size	Age mean/median(year)	Men proportion (n, %)	Measurement of Covid-19*	Obesity Prevalence (n, %)	Diabetes Prevalence (n, %)	Hypertension Prevalence (n, %)	Hyper lipidemia Prevalence (n, %)
1	Alattar R, et al. [18]	Qatar, 2020	Retrospective Cohort	Local	25	58(50–63)	23(92%)	1	-	12(48%)	-	-
2	Barrasa H, et al. [9]	Spain, 2020	Cohort	National	48	63 (12)	27 (56%)	5 & 1	23 (48%)	9 (19%)	21 (44%)	-
3	CDC COVID-19 Response Team [19]	USA, 2020	Cross-Sectional	National	7162	-	-	1	-	10.9%	-	-
4	Claire E Hastie, et al. [20]	England, Scotland and Wales, 2020	Cross-Sectional	Regional	449	(37–73)	256(59.02%)	1	158 (35.19%)	400 (89.09%)	-	-
5	Gentile S, et al. [5]	Italy, 2020	Cross-Sectional	National	1890	-	-	1	12.2%	31.9%	69.7%	-
6	GuanW J, et al. [21]	China, 2020	Retrospective	National	1590	48.9(16.3)	904(57.3%)	1	-	8.2%	16.9%	-
7	Guo W, et al. [22]	China, 2020	Retrospective	Local	174	59 (49–67)	76 (43.7%)	1,2	-	37(21.2%)	43 (24.7%)	-
8	Hong K S, et al. [23]	South Korea, 2020	Retrospective Cohort	Local	98	55.4(17.1)	38(38.8%)	1	-	9(9.2%)	30(30.6%)	-
9	Nikpouraghdam M, et al. [24]	Iran, 2020	Retrospective	Local	2964	55.50 (15.15)	1955(66%)	1	-	113(3.81%)	59(1.99%)	-
10	Sean Wei Xiang Ong, et al. [6]	Singapore, 2020	Retrospective Cohort	Local	91	(NM-60)	-	1	12.1%	18(19.8%)	30(33%)	-
11	Shi, Y, et al. [25]	China, 2020	Retrospective Cohort	Local	487	46 (19)	259 (53.2%)	2 & 3 & 4	-	29 (6.0%)	99 (20.3%)	-
12	Simonnet A, et al [26]	France, 2020	Retrospective Cohort	Local	124	60(51–70)	73%	1 & 3	47.6%	28 (23%)	60 (49%)	34 (28%)
13	Yang zhang, et al. [7]	China, 2020	Retrospective	Local	166	62.7(14.2)	85(51.2%)	3,4	-	21.1%	76(45.8%)	-

*measure type (PCR: 1, CT: 2, clinical: 3, Lab: 4, World Health Organization interim guidance by RT-PCR: 5)

All included studies were retrospective in design (Cohort or Cross-sectional). The diagnosis of Covid-19 was based on World Health Organization’s interim guidance in all studies (by RT-PCR). These studies were the ones reported on the prevalence of metabolic risk factors (diabetes, obesity, and hypertension).

A total sample of 2,602, 7,632, and 15,268 patients with Covid-19 was included in our analysis of obesity, diabetes, and hypertension, respectively. There was not any study that specifically focused on MetS. The prevalence of hyperglycemia was reported only through one paper (28%).

The mean age for diabetes and hypertension analysis based on what reported (from only six studies) was 55.25 years and about 61% of patients were males in all three analysis. The maximum number of cases was 7,162 and belongs to the USA, whereas the minimum was 91 and belongs to Singapore.

The prevalence of diabetes was reported on all 13 studies while 10 of them reported the patient’s hypertension prevalence and only 5 of them reported the patient’s obesity prevalence. This prevalence was not reported in terms of sex or age groups so we were not able to perform sub-group analysis.

### 3.1. Prevalence of obesity in Covid-19 patients

**Fig 2:**
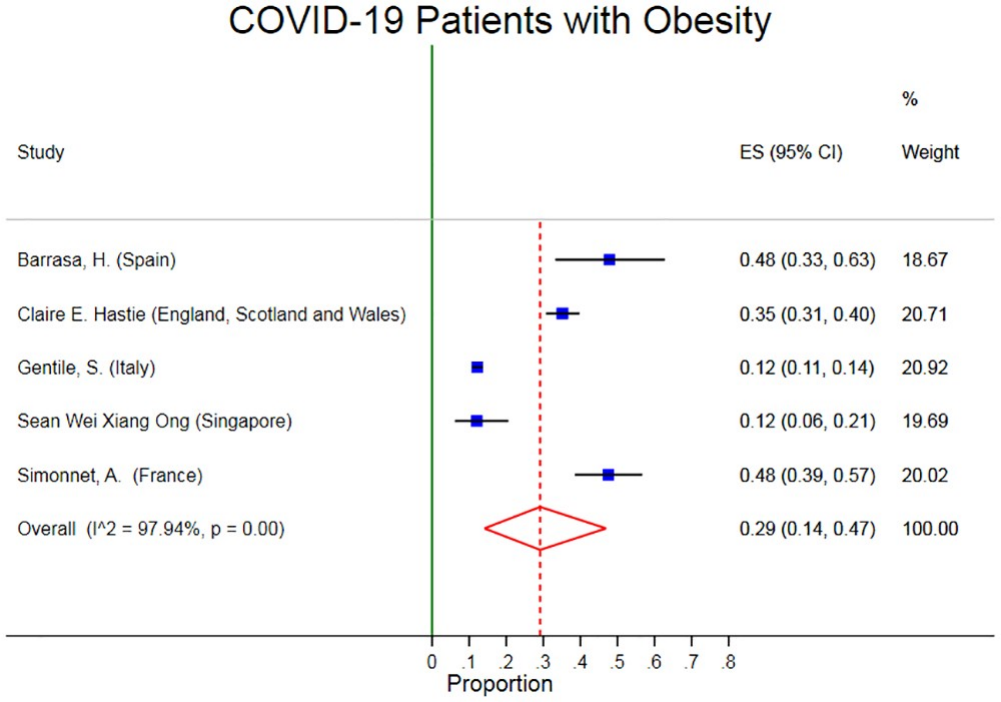
Forest plots of pooled prevalence of obesity in Covid-19 patients.

The pooled prevalence of Obesity was 29% (95% CI: 14–47%). There was obvious heterogeneity (I2: 97.94%, p < 0.001) in the prevalence of obesity in these studies (Fig 2). The Egger’s test p-value was 0.19 which means there is no indication of small-study effects.

**Fig 3:**
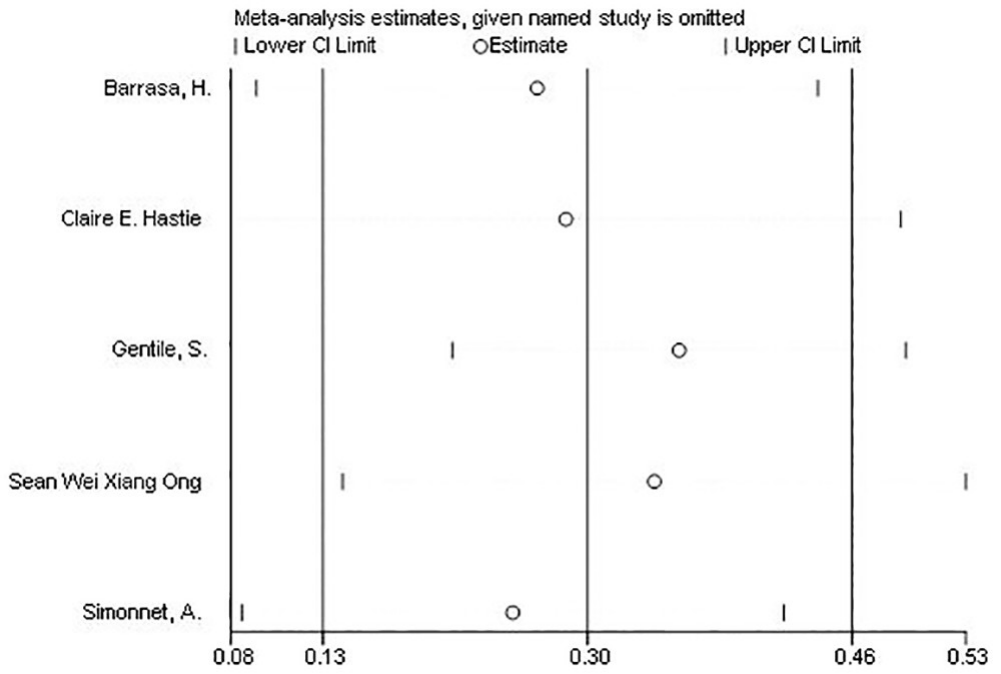
Forest plots of sensitivity analyses of each included study in meta-analysis of the prevalence of obesity in Covid-19 patients.

Several sensitivity analyses were done to test the robustness of the observed association. Elimination of any single study at a time from the meta-analysis did range from 25% (95% CI: 8.4%-42%) to 35% (95% CI: 21%-49%) that indicate moderate robustness of the pooled estimates of prevalence (Fig 3).

### 3.2. Prevalence of diabetes in Covid-19 patients

**Fig 4:**
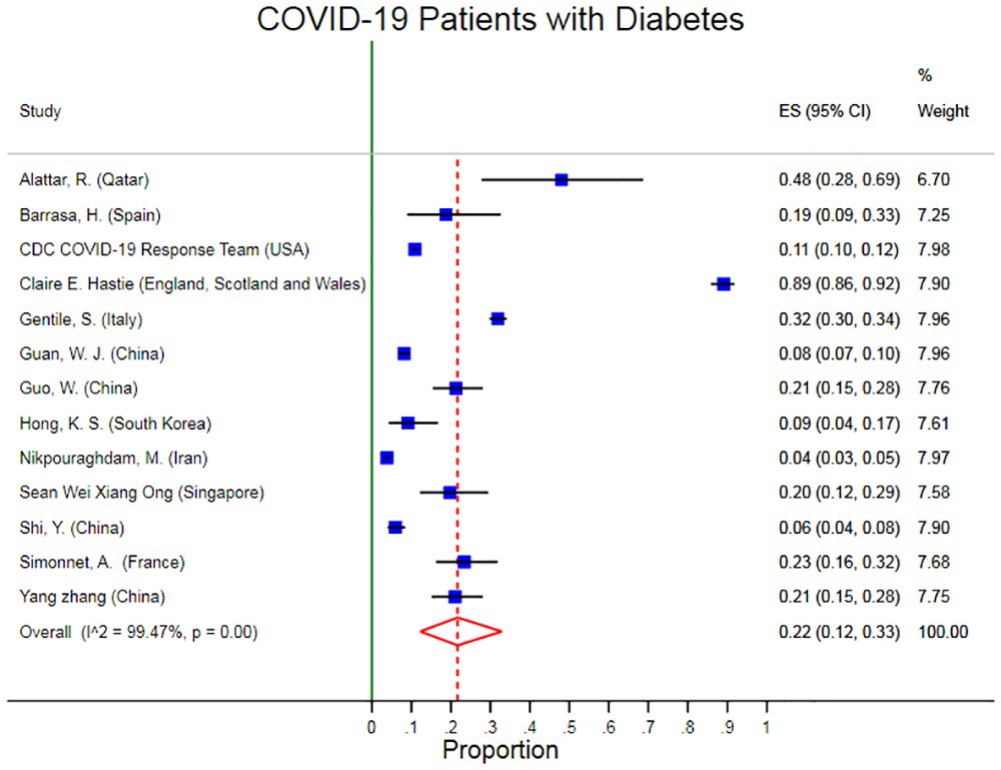
Forest plots of pooled prevalence of diabetes in Covid-19 patients.

The pooled prevalence of Diabetes was 22% (95% CI: 12% 33%). There was obvious heterogeneity (I2: 99.47%, p < 0.001) in the prevalence of Diabetes in these studies (Fig 4). The Egger’s test p-value was 0.27 which means there is no indication of small-study effects.

**Fig 5:**
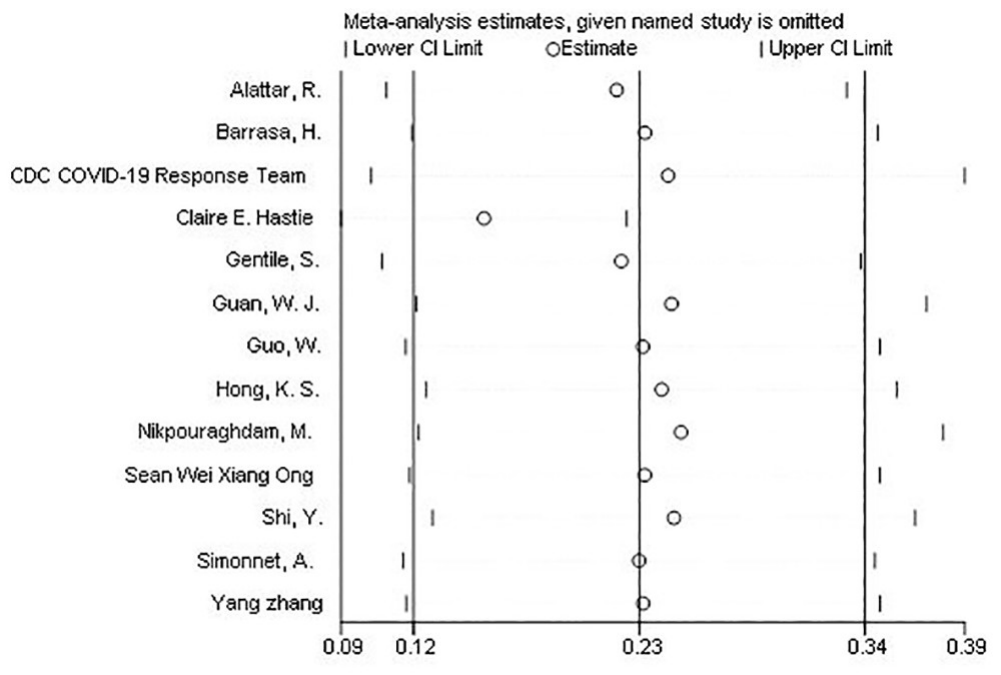
Forest plots of sensitivity analyses of each included study in meta-analysis of the prevalence of diabetes in Covid-19 patients.

Several sensitivity analyses were conducted to test the robustness of the observed association. The exclusion of any single study at a time from the meta-analysis did range from 22% (% CI: 11%-33%) to 25% (95% CI: 13%-38%) that indicate very high robustness of the pooled estimates of prevalence (Fig 5).

### 3.3. Prevalence of hypertension in Covid-19 patients

**Fig 6:**
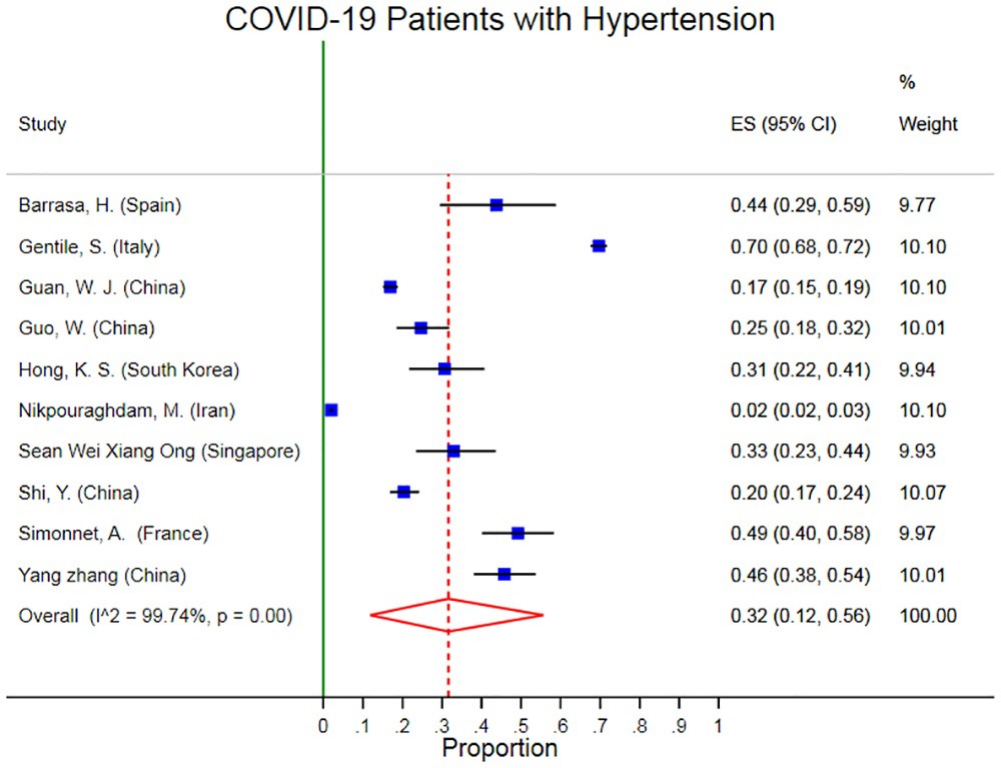
Forest plots of pooled prevalence of hypertension in Covid-19 patients.

The pooled prevalence of hypertension was 32% (95% CI 12% 56%). There was obvious heterogeneity (I2 99.74%, p < 0.001) in the prevalence of obesity in these studies (Fig 6). The Egger’s test p-value was 0.63 which means there is no indication of small-study effects.

**Fig 7:**
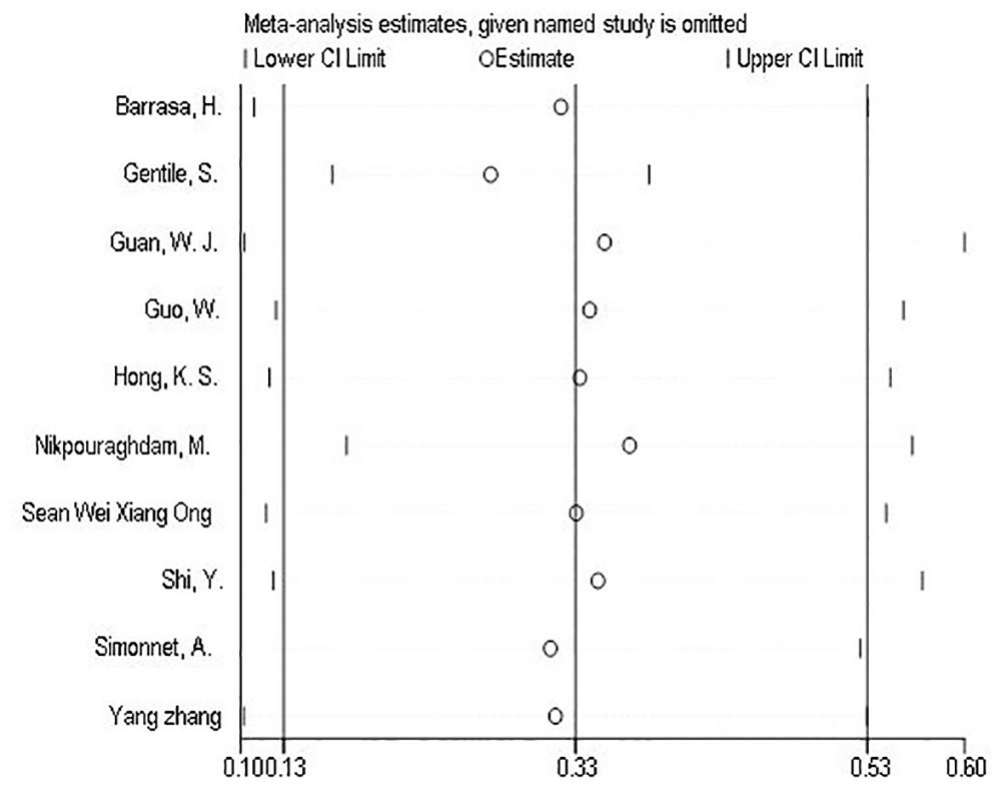
Forest plots of sensitivity analyses of each included study in meta-analysis of the prevalence of hypertension in Covid-19 patients.

Several sensitivity analyses were conducted to test the robustness of the observed association. The exclusion of any single study at a time from the meta-analysis did range from 27% (% CI 17%-38%) to 37% (95% CI 18%-56%) that indicate moderate robustness of the pooled estimation of prevalence (Fig 7).

The results showed the estimated pooled prevalence of obesity, diabetes, and hypertension was 29%, 22%, and 32% respectively. So, the pooled prevalence of hypertension is the highest and the pooled prevalence of diabetes is the lowest ones. This means, in other words, that hypertension is more prevalent than obesity and diabetes in patients with Covid-19 infection.

## 4. Discussion

To the best of our knowledge present investigation as one of the pioneers’ comprehensive analytical studies on the association of Metabolic risk factors and risk of Covid-19, summarized practical findings of related evidence and provide a wide range of information to policymakers, clinical specialists, researchers, and other stakeholders.

Present meta-analysis has collected data from all observational studies on Covid-19 patients with obesity/overweight, diabetes mellitus, hypertension, and dyslipidemia in the world. The diagnosis of SARS COV-2 was by RT-PCR (based on the World Health Organization interim guidance).

Consistent with previous reports, we found a considerable prevalence of metabolic risk factors, which most likely act as a risk factor, in COVID-19 patients. The pooled prevalence of obesity in Covid-19 patients was 29% (95% CI: 14–47%). For Diabetes and hypertension these were 22% (95% CI: 12% 33%) and 32% (95% CI 12% 56%), respectively. This indicates that hypertension (32%) is more prevalent than obesity (29%) than diabetes (22%) in patients with Covid-19 infection.

There were significant and high levels of heterogeneity (more than 95%) for the pooled prevalence of obesity, diabetes, and hypertension. The existing heterogeneity could be partly due to sampling size, design, the study scope, screening methods, and diagnostic method, which was not solved by our sensitivity analysis.

In the sensitivity analysis, the exclusion of any study did not have a significant impact on the pooled estimates, which displayed a rather low sensitivity. Our guess is that a variety of study scope (national, regional, and local) have led to heterogeneity. However, due to the scarce number of studies, we could not check it through a meta-regression analysis.

Yet, we can rely on these estimates, as the small study effects that were checked by Egger’s test showed no significant indication of any effects on the pooled prevalences.

Based on the raised probabilities, metabolic risk factors could be associated with the possibility of infection and even the severity of symptoms and consequences of the Covid-19 disease [9, 25, 27].

It is mentionable that; in early clinical reports, obesity/ overweight was rarely noticed among the significant clinical risk factors for Covid-19. Complementary findings emphasized on a high frequency of obesity among patients admitted to intensive care for Covid-19 disease [26, 28–30].

In line with the findings of the present study, a previous study discussed that patients with T2DM are susceptible to infections of the lower (but not upper) respiratory system [31]. People with T2DM are more susceptible to Covid-19 infection, with twice as high mortality risk as metabolically healthy people [5]. Another evidence revealed that compared with non-diabetic patients, patients with diabetes are more likely to be admitted to hospital [31, 32]. Some related studies have also emphasized the role of hypertension as a risk factor of Covid-19 disease [33, 34].

Investigate the possible causes of these associations; exceed weight has been described as an independent predisposition factor for severe pulmonary infections [35]. Besides, abdominal obesity results in reduced blood oxygen saturation through impairing the processes of ventilation, especially at the base of the lungs [6]. As another confirmed reason, the abnormal secretion of adipokines and cytokines characterize a chronic low-grade inflammation situation of abdominal obesity, that could impair immune responses [7].

It is still unknown whether obesity or diabetes acts as a risk factor for the prognosis of COVID-19. As the most likely mechanism T2DM is widely proposed as a chronic, low-grade inflammatory disease caused by an imbalance of long term immune system, metabolic syndrome, or nutrient excess associated with excess weight and obesity. Obesity-associated inflammation, which characterized by increasing abundance and activation of innate and adaptive immunity cells in adipose tissue along with an increased release of inflammatory factors and chemokines locally and systemically [22, 36].

Hypertension studied as a host risk factor for Covid-19 may underscore the involvement of the renin-angiotensin system (RAS) in the pathogenesis of the disease [33, 34].

It has been accepted that through a complex setting, some comorbidities frequently coexist. For instance, diabetes frequently coexists with hypertension, obesity, or even coronary heart diseases [21, 23]. In these situations, patients with coexisting metabolic risk factors are more likely to be at risk of debases and poorer outcomes [21].

Practically based on the recommendations of most studies considering the variations of patterns and different trends in a different population [5], Patients with cardiometabolic risk factors and especially those with multiple risk factors should take extra measures to avoid Covid-19 contamination by enforcing [26]. In this regard further studies, particularly those enrolling different participants from various groups, are needed to validate the practical findings [25].

The present investigation has some strengths and limitations. This attempt was the first comprehensive systematic review and meta-analysis on the association of metabolic risk factors and Covid-19 disease. Most of the included studies were benefitting from the retrospective cohort design provide related data from interactive new databases. In all papers diagnosis of Covid-19 was based on World Health Organization interim guidance in all studies (by RT-PCR).

As one of the mentionable limitations, as like any other secondary study, the quality of our data was dependent on the accuracy and quality of the data reported in the primary studies. Due to the limited number of studies and heterogeneity of reported specification we were not able to analyze the data in subgroups of sex, age, ethnicity, and other practical fields. As another hint, many potential factors such as physical activity, smoking, alcohol consumption, job description, and education, were not controlled.

As the most important implications; results of present meta-analysis reveal important information for both; policymakers and the general population to protect them against the confirmed risk of disease. This report showing the prevalence of metabolic risk factors in patients with Covid-19, which is beneficial to management and inhibit the spread of disease in the future. As the main suggestions:

- Co-occurrence of metabolic risk factors in suspected individuals or definite cases of Covid-19 require close cooperation between different specialists in order to carry out an appropriate clinical evaluation process for each individual as soon as possible.
- Given the limitations of diagnostic capacities and medical services, as well as the possibility of imminent production of vaccines or more approved therapies, it is necessary to plan for prioritizing the availability of these groups and the allocation of future facilities.
- Special attention should be paid to this group of COVID-19 patients as they may suddenly develop acute heart complications or problems that alter their recovery.
- Proper triage of these groups of patients based on a comprehensive evaluation of metabolic risk factors provides appropriate supporting plan through which prompt protective procedure follows by health providers and patients themselves.
- Another important point is to pay attention to the accurate collection and recording of data that can help analyze the health status of these groups and allow them to be followed up after diagnose/ discharge.
- Cardiometabolic prevention must be considered as the most important priority through the post-pandemic recovery phase of COVID-19. These require significant investment in interventions addressed health-promoting and lifestyle promotion.
- In addition, more activities should be made to compensate for the above-mentioned limitation in future studies.

## 5. Conclusion

In summary, by identifying host risk factors associated COVID-19 this study shed light on the underlying mechanisms of disease progression. The results may help in the promotion of patient management while helping to develop better policies for prevention and response to COVID-19 and its critical outcomes. The prevalence of co-morbid metabolic risk factors must be adopted for better management and priority settings of public health vaccination and other required interventions. Considering the evident gap in related evidence, more adequately powered investigations should be run to assess the associations.

## Supporting information

S1 File (XLSX)

S1 Checklist PRISMA 2009 checklist. (DOCX)
